# Analysis of Pre-Driver and Last-Stage Power—Ground-Induced Jitter at Different PVT Corners

**DOI:** 10.3390/s22176531

**Published:** 2022-08-30

**Authors:** Malek Souilem, Rui Melicio, Wael Dghais, Hamdi Belgacem, Eduardo Rodrigues

**Affiliations:** 1 École Nationale d’Ingénieurs de Sousse, Université de Sousse, Sousse 4054, Tunisia; 2 Laboratoire d’Electroniques et Microélectroniques, Université de Monastir, Monastir 5000, Tunisia; 3 IDMEC, Instituto Superior Técnico, Universidade de Lisboa, 1049-001 Lisboa, Portugal; 4 ICT—Instituto de Ciências da Terra, Universidade de Évora, Rua Romão Ramalho 59, 7000-671 Évora, Portugal; 5 Institut Supérieur des Sciences Appliquées et de Technologie de Sousse, Université de Sousse, Sousse 4003, Tunisia; 6 INESC-ID, Sustainable Power Systems Group, Instituto Superior Técnico, Universidade de Lisboa, Av. Rovisco Pais 1, 1049-001 Lisboa, Portugal

**Keywords:** PVT corner analysis, eye diagram jitter, power supply-induced jitter, power delivery network, IBIS algorithm, power and ground noise, signal and power integrity, aerospace

## Abstract

This paper presents the study of power/ground (P/G) supply-induced jitter (PGSIJ) on a cascaded inverter output buffer. The PGSIJ analysis covers the IO buffer transient simulation under P/G supply voltage variation at three process, voltage, and temperature (PVT) corners defined at different working temperatures and distinct P/G DC supply voltages at the pre-driver (i.e., $V_{DD}$/$V_{SS}$) and last stage (i.e., $V_{DDQ}/V_{SSQ}$). Firstly, the induced jitter contributions by the pre-driver, as well as the last, stage are compared and studied. Secondly, the shared and decoupled P/G supply topologies are investigated. The outcomes of these simulation analyses with respect to worst case jitter corners are determined, while highlighting the importance of modeling the pre-driver circuit behavior to include the induced jitter in the input–output buffer information specification (IBIS)-like model. Accordingly, the measured PGSIJ depends on the corners to be analyzed and, therefore, the designer needs to explore the worst-case corner for the driver’s technology node and the most supply voltage noise affecting the jitter output for signal and power integrity (SiPI) simulations. Finally, the jitter transfer function sensitivity to the amplitude and frequency/phase variations of the separate and combined impacts of the pre-driver and last stage are explored, while discussing the superposition of the power supply induced jitter (PSIJ) induced by both the driver’s IO stages under small signal and large signal supply voltage variations. The linear superposition of the separate PSIJ effects by the pre-driver and last stage depends on the amplitude of the variation of the supply voltage that can drive the transistor to their nonlinear working regions.

## 1. Introduction

The assessment of signal integrity and power integrity (SiPI) of mixed signal interconnected digital input–output (IO) link aims to simulate the impairments of the channel and power delivery network (PDN), respectively. Therefore, it is important to explore SiPI assessment at different process, voltage, and temperature (PVT) corners of the designed IO link in order to ensure a good quality of the signal propagating on the package and printed circuit board (PCB) interconnects, hence a robust IO link design [1,2,3]. In fact, several industries (e.g., automotive, aerospace, consumer electronics) are driving the need for power integrity and PDN analysis due to the high density of multichip design and the massive high-speed data processing requiring fast DDR memory, server CPU, and multi-level signaling with low-level power rail voltages for power saving [2,3].

Timing and amplitude distortions of the signals (i.e., currents and voltages) are not only due to channel design, such as inter-symbol interference, reflection, and crosstalk between lines, but can also be induced by the power and ground supply voltage (PGSV) variations at the die level. In fact, the fast and power state-dependent switching current profile of different power and ground supply domains lead to a considerable power and ground supply noise, e.g., (L.di/dt) that affects the nonlinear dynamic electrical behavior of the transistor-based IO device. Consequently, it is crucial to study the IO device’s SiPI issues under different PVT corners and supply voltage variations, input signal noise, and crosstalk, etc., in order to ensure a robust and reliable device performance [1,2].

**Figure 1:**
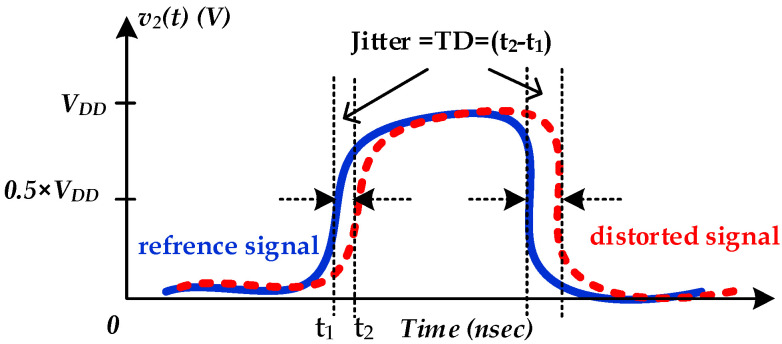
Illustration of timing distortion known as jitter occurring at the rising and falling transition of the output signal. Reference signal (solid blue line). Distorted signal (red dashed line).

In fact, jitter is defined by the timing deviation (e.g., $\mathrm{TD}=t_{2}-t_{1}$) of the distorted output signal (e.g., assuming PGSV variations) under a sequence of transition edges from their ideal positions (e.g., with constant DC PGSV), as shown in Figure 1. These timing differences depend on the PGSV variations and can be different for the rising and falling transitions [4], under a determined bit error rate and data speed. Therefore, designers need to estimate the jitter induced by SiPI simulation of high-speed IO links [5,6].

**Figure 2:**
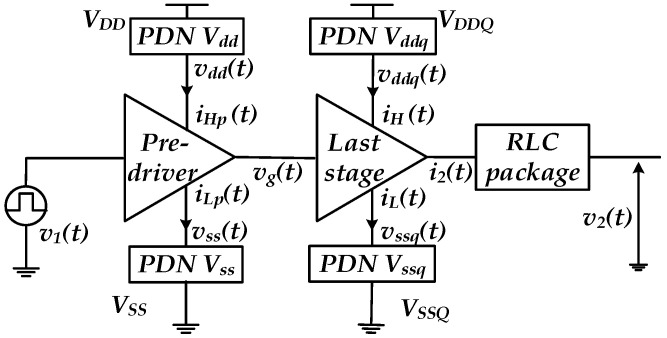
IO buffer circuit diagram with separate PDN for the pre-driver and last stage.

Furthermore, PGSV variations affect the transistor current–voltage (I–V) and capacitance–voltage (C–V) operation regions (e.g., cut-off, linear and saturation) forming the output buffer/driver [5]. Moreover, distinct PDNs are commonly deployed for powering the pre-driver and last stage, $V_{DD}/V_{SS}$ and $V_{DDQ}/V_{SSQ}$, respectively, as shown in the IO buffer circuit diagram of Figure 2. The device’s switching currents in the last stage (e.g., $i_{H}\left(t\right)$ and $i_{L}\left(t\right))$ and pre-driver stage (e.g., $i_{Hp}\left(t\right)$ and $i_{Lp}\left(t\right))$, flowing via the PDN, produce the PGSV variations, generating the timing distortions at the output voltage, which is defined by the P/G supply-induced jitter (PGSIJ) [7].

For example, $h_{vdd}\left(t\right)$ and $h_{vddq}\left(t\right)$ are defined as the impulse response of the pre-driver and last stage’s off-die PDN. Moreover, $I_{Hp}\left(t\right)$ and $I_{H}\left(t\right)$ are the switching current of the pre-driver and last stage IO buffers, as shown in Figure 2. Therefore, the power supply voltage (PSV) ripples induced at the on-die pre-driver and buffer’s last stage can be written in the time and frequency domains in Equation (1). The equivalent frequency domain voltage noise is formulated as a multiplication of the current spectrum and the PDN impedance $Z_{PDN}$

(1) $$\{\begin{array}{c} v_{dd}\left(t\right)=\int_{0}^{+\infty }\;h_{vdd}\left(t-\tau \right)\cdot I_{Hp}\left(\tau \right)\;d\tau \;\Leftrightarrow V_{DD}\left(f\right)=Z_{vdd}^{PDN}\left(f\right)\times I_{Hp}\left(f\right)\\ v_{ddq}\left(t\right)=\int_{0}^{+\infty }\;h_{vddq}\left(t-\tau \right)\cdot I_{H}\left(\tau \right)\;d\tau \;\Leftrightarrow V_{DDQ}\left(f\right)=Z_{vddq}^{PDN}\left(f\right)\times I_{H}\left(f\right) \end{array}\;$$

Similar to Equation (1), it can be derived for the ground supply voltage variations

(2) $$\{\begin{array}{c} v_{ss}\left(t\right)=\int_{0}^{+\infty }\;h_{vss}\left(t-\tau \right)\cdot I_{Lp}\left(\tau \right)\;d\tau \;\Leftrightarrow \;V_{SS}\left(f\right)=Z_{vss}^{PDN}\left(f\right)\times I_{Lp}\left(f\right)\\ v_{ssq}\left(t\right)=\int_{0}^{+\infty }\;h_{vssq}\left(t-\tau \right)\cdot I_{L}\left(\tau \right)\;d\tau \;\Leftrightarrow V_{SSQ}\left(f\right)=Z_{vssq}^{PDN}\left(f\right)\times I_{L}\left(f\right) \end{array}\;$$

The PDN characteristics in terms of the resonance frequency, bandwidth, loop inductance, etc. (i.e., $Z_{vdd}^{PDN}\left(f\right)/Z_{vss}^{PDN}\left(f\right)\;$and $Z_{vddq}^{PDN}\left(f\right)/Z_{vssq}^{PDN}\left(f\right))$), and switching current spectrum (i.e., $I_{Hp}\left(f\right)$/$\;I_{Lp}\left(f\right)$ and $I_{H}\left(f\right)/\;I_{L}\left(f\right)$) are different for both $V_{DD}/V_{SS}$ and $V_{DDQ}/V_{SSQ}$. Therefore, the jitter/timing distortions induced by the IO buffer pre-driver and the last-stage PGSV variations present different mechanisms and performance numbers (e.g., [3,6]).

Previous IO buffer-modelling methodologies, which can be classified either based on the equivalent circuit input–output buffer information specification (IBIS) or parametric curve fitting [8,9], have not clearly addressed the PVT corner simulation and its importance in the model’s generation steps, worst corner identification, and in the validation of model performance. In fact, the standard multiport behavioral model structure that describes the electrical behaviors of the IO buffer circuit, while considering the PGSV variables [7,8,9], is:

(3) $$i_{2}\left(t\right)=w_{L}\left(t\right)F_{L}\left(x_{dd}\left(t\right),\frac{dx_{dd}}{dt}\right)+w_{H}\left(t\right)F_{H}\left(x_{ss}\left(t\right),\frac{dx_{ss}}{dt}\right)+i_{ddq}\left(t\right)+i_{ssq}\left(t\right)$$

where voltage differences${\;x}_{dd}\left(t\right)=v_{dd}\left(t\right)-v_{2}\left(t\right)$ and $x_{ss}\left(t\right)=v_{2}\left(t\right)-v_{ss}\left(t\right)$ are applied to the $F_{L}\left(\cdot \right)$ and $F_{H}\left(\cdot \right)$ functions that model the nonlinear dynamic output admittances of the driver’s last stage under low and high input logic levels, respectively. This model considers not only the static contribution of the PG voltage fluctuation of the last stage, but also the delay introduced by the pull-up (PU) and pull-down (PD) capacitances, which are represented by the derivatives.$\;i_{ddq}\left(t\right)$ and $i_{ssq}\left(t\right)$ are timing current (I-*t*) tables, that include crow-bar, on-die decoupling, pre-driver, and current contributions, which are provided by the IBIS power-aware enhancement to predict the voltage ripple introduced at the IO buffer’s circuit supplied by $V_{DDQ}$, [8,9,10,11,12,13,14,15,16]. $w_{L}\left(t\right)$ and $w_{H}\left(t\right)$ are switching timing signals that capture the IO timing behavior of the pre-driver stage, which is powered by $V_{DD}/V_{SS}$ supplies.

Since these supplies are not constant due to high-current switching through the pre-driver’s PDN, therefore, the IBIS model fails to accurately predict the timing distortion originating from $V_{DD}$, the voltage noise which affects the output eye jitter. Moreover, previous works presented an extended equivalent circuit behavioral model for SiPI simulation in the pre-driver and the driver’s last stage [10,11,12]. Moreover, ground supply noise has not been considered [12]. For these reasons, this paper explores the study of the separate effect of the jitter distortion induced by both stages powered by distinct P/G supplies. For instance, the methodology consists of analyzing the jitter distortion induced by the IO buffer’s circuit stages under three PVT corners with different circuit P/G supply configurations or scenarios which cover most of the practical IO buffer design with a separate PDN design. The analysis carried out in this work is based on transient simulation of the IO buffer transistor level (TL) spice reference model, at three main PVT corners with different configurations of the PGSV variations connected at the pre-driver and last stage. 

The rest of the paper is organized as follows. Section 2 describes power supply induced jitter (PSIJ) prediction methodology followed in this paper. Section 3 presents the IO buffer circuit along with corner definition and circuit configuration to be simulated and analyzed. Moreover, it describes the jitter analysis of the pre-driver and last stage contributions, along with the shared vs. decoupled PGSV configuration. Section 4 investigates the PSIJ transfer function dependency on the frequency and amplitude variations of the driver’s IO stages. Conclusions and future work are presented in Section 5.

## 2. Methodology: PSIJ under PVT Corners and Jitter Sensitivity

**Figure 3:**
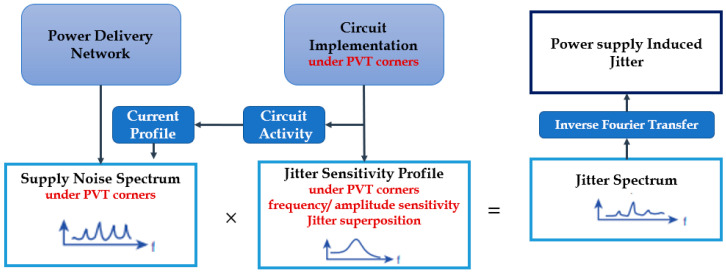
Block diagram illustrating the variables contributing to PSIJ generation.

In order to accurately predict the PSIJ by the IO circuit, it is crucial to provide the accurate model representation of the PDN, the current switching due to data pattern activity and the jitter sensitivity. The combinations of these three inputs are illustrated in Figure 3, which presents the generic frequency modelling approach of the PSIJ in the frequency domain. In fact, the resulting supply noise spectrum (i.e., $V\left(f\right)=Z_{PDN}\;\left(f\right)\times I\left(f\right)$ from Equations (1) and (2)), is multiplied by the jitter sensitivity, S(f), $\left[\mathrm{ps}\;{\mathrm{mV}}^{-1}\right]$, to yield to the jitter spectrum J(f).

(4) J(f)=V(f)×S(f) 

The examples of the PDN impedance profile modeling methodologies and extraction are shown in [17,18]. Current switching profiles can be simulated based on transient simulation or estimated at the early design stage [18]. The noise-to-jitter transfer function sensitivity is determined by characterizing the IO buffer under PGSV noise and measuring the output eye jitter.

It is worth noting that the jitter sensitivity profile only depends on the intrinsic transistor-based circuit implementation of the IO buffer. Once the jitter spectrum is obtained, the time domain jitter is formed by applying an inverse of Fourier transform to determine the jitter in time domain. This methodology can be applied both in the pre-layout and/or post-layout phase of the circuit and PDN design because it helps the system on chip (SoC) design team to figure out the necessary on-die and package decoupling capacitor requirements, along with PCB/package inductance, leading to an acceptable supply noise profile and jitter that can be tolerated by their system.

Since a distorted sinusoidal waveform is typically induced at the PGSV, the jitter sensitivity function to supply noise frequency, S(f), can be determined via transient simulation by sweeping the frequency of the voltage noise over the frequency range of interest at which the device is more sensitive to the jitter. Nevertheless, the derivation of the simulation data and setup is time consuming and requires high computational resources.

Another theoretical approach that has led to an analytical approximate solution for open-loop circuit paths is proposed to model the frequency-dependent sensitivity of jitter by characterizing the IO buffer delay difference at two different bias voltages. *S*(f) can be identified straightforwardly by the following analytical method [19,20].

(5) $$\{\begin{array}{c} S\left(f\right)=\frac{j}{2\pi f\tau_{d}}S_{0}\left[1-e^{-j2\pi f\tau_{d}}\right]\\ S_{0}=\frac{TD_{V1}-TD_{V2}}{V_{2}-V_{1}} \end{array}\;$$

$S_{0}$ is the DC delay sensitivity, which is determined by the static delay difference of the $TD_{V1}$ and $TD_{V2}$ at their respective dc voltages, $V_{1}$ and $V_{2}$, respectively, as illustrated in Figure 1. $\tau_{d}$ is the sub-circuit path delay. Therefore, the jitter sensitivity magnitude can be expressed as follow:

(6) $$\left|S\left(f\right)\right|=S_{0}\left|\frac{\sin\left(\pi f\tau_{d}\right)}{\pi f\tau_{d}}\right|=S_{0}\left|sinc\left(\pi f\tau_{d}\right)\right|\;$$

The jitter prediction methodology in the frequency domain of Figure 3, which is either based on simulation data or the analytical theoretical approximation of the PSIJ sensitivity, assumes that the supply ripple noise is regarded as perturbation and thus it is behaving as a small signal with respect to the operation point of the driver’s circuit IO stages (i.e., pre-driver and last stage). Therefore, the jitter sensitivity is assumed to be independent of the supply noise amplitude. However, the PSIJ flow and jitter sensitivity cannot be considered as a linear-time-invariant system because the validity range of the linear approximation depends on supply voltage and IO buffer PVT corners [20]. For this reason, this paper explores:

- The consideration of PVT corners to analyze the separate contribution of PGSIJ in the pre-driver or last-stage circuits and their combined PGSIJ contribution, as both driver’s IO stages can share the same PDN or have a distinct PDN design, where the decoupled PG supply noise can have different noise waveforms.
- The derivation of experimental frequency- and amplitude-dependent jitter transfer functions of the pre-driver and last stage IO buffer circuits from transient TL circuit simulation under the worst-case corner determined from the above first analysis. Moreover, the two-tone jitter superposition validity under small and large supply voltage variations for both of the driver’s stages is studied and analyzed.

## 3. Jitter Analysis under PVT Corners: Simulation Setups and Results

### 3.1. IO Buffer Circuit and PVT Corners

**Figure 4:**
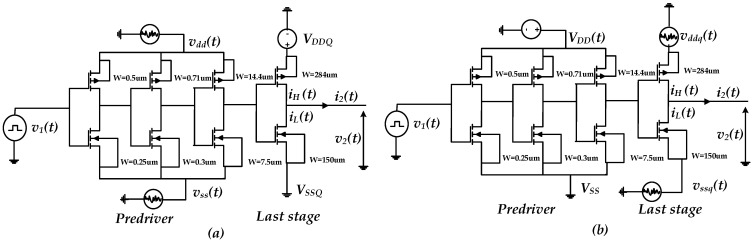
Simulation setup used to evaluate the impact of PGSV variations independently connected at the (**a**) pre-driver or (**b**) driver’s last stage.

**Figure 5:**
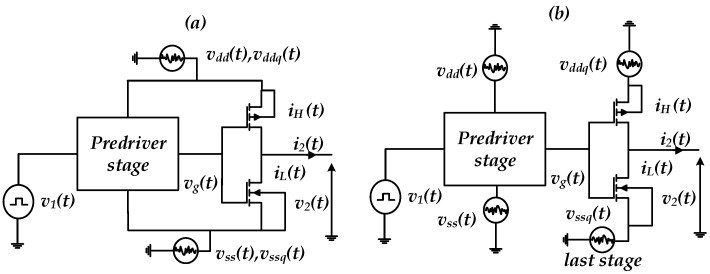
Simulation setups used to evaluate the p2p eye jitter. (**a**) two-tone voltage noise applied to the shared PG rails. (**b**) two-tone voltage noise applied the decoupled PG rails.

The considered driver circuits with their respective PDNs for PGSIJ analysis and evaluation are shown in Figure 4. The driver is composed of four cascaded inverters in series and designed in 0.35 µm technology. The nominal supply voltage ${\left(V_{DDQ}/V_{DD}\right)}_{Nom}=3.3\;V$. The first three inverters combinedly represent the pre-driver stage, whereas the last stage is composed of the front-end inverter. The driver’s output-induced jitter is investigated by estimating the pre-driver and last-stage jitter contributions independently. Then, the jitter induced by the shared and decoupled PGSV configurations are also studied, as shown in Figure 5. This work assumes that PGSV are the sum of sinusoidal voltage sources which are applied around a dc voltage. The IO buffer is simulated with a 500 Mbps input data rate at different corners. Each corner has a specific level of $V_{DD}/V_{DDQ}$ and a defined temperature: slow–slow (SS), typical–typical (TT), and fast–fast (FF) corners as described in Table 1.

In the decoupled case, the pre-driver is powered by $v_{dd}\left(t\right)=V_{DD}^{corner}+v_{dd}^{noise}\left(t\right)$ and $v_{ss}\left(t\right)=V_{ss}^{noise}\left(t\right)$. Moreover, the last stage is powered by $v_{ddq}\left(t\right)=V_{DDQ}^{corner}+v_{ddq}^{noise}\left(t\right)$ and $v_{ssq}\left(t\right)=v_{ssq}^{noise}\left(t\right)$. In the shared case, P/G terminals of the pre-driver and last stage are shorted and a single voltage noise is used at the P/G supplies. The peak-to-peak (p2p) AC noise applied to the PGSV is defined to be within 20% of ${\left(V_{ddq}/V_{dd}\right)}_{Nom}$ (e.g., 660 mVpp).

### 3.2. Induced Jitter: Pre-Driver vs. Last Stage

This study carried out an experiment to investigate the impact of the separate PGSV variations at the pre-driver or the last-stage supplies on the output jitter, as shown in Figure 4. Firstly, two different signal tones are applied to the pre-driver power supply $v_{dd}\left(t\right)$ and ground supply, $v_{ss}\left(t\right)$. These voltage sources are applied on the dc voltage for each corner, while the last stage is biased with a constant dc voltage source, as shown in Figure 4a. Moreover, the same noise sources described above are used at the driver’s last-stage terminals, $v_{ddq}\left(t\right)$ and $v_{ssq}\left(t\right)$, while the pre-driver stage is biased by constant dc sources, as presented in Figure 4b. Table 2 presents the different noise parameters, frequency, and amplitude values used in this setup.

The simulation results of the p2p eye jitter, along the with eye width (EW) and eye height (EH) openings, are reported in Table 3. Eye measurements are determined between a 40% and 60% eye boundary time crossing and 20% and 80% of the amplitude thresholds, respectively. This result clearly shows that, for this IO buffer topology, the PGSIJ difference between the pre-driver and last stage is ~10 ps at the slow–slow (SS−40 °C) corner (($V_{DD}=V_{DDQ\;}$= 2.97 V, T = −40 °C). Therefore, the design engineer should consider if this analysis is carried out by means of behavioral models extracted or generated based on the IBIS or its power-aware version that shows several shortcomings in capturing PGSIJ by the pre-driver’s supply $V_{DD}/V_{SS}$ domains, because they are kept constant during device characterization and model extraction [14,15].

**Figure 6:**
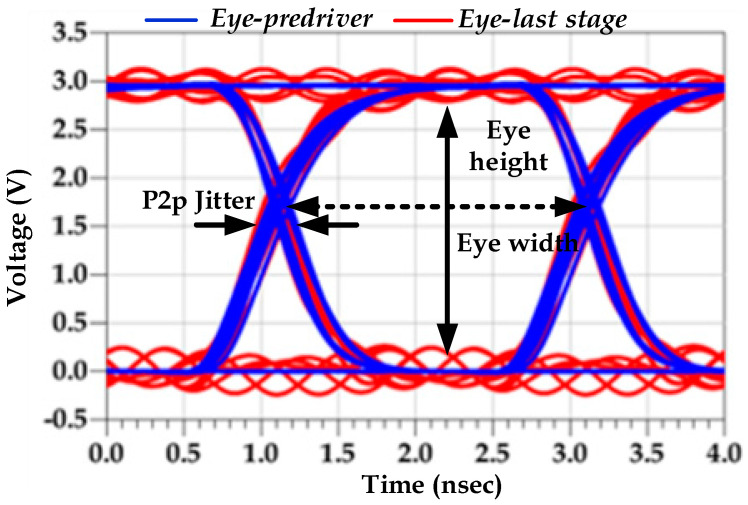
Eye diagram: pre-driver vs. last-stage PGSIJ at the SS−40 °C corner.

The numerical results of the p2p eye jitter, which are reported in Table 3, confirm that the pre-driver PGSIJ can be as important as last-stage jitter. The percentage of the jitter distortion with respect to the unit interval (UI = 1/data rate) is added to demonstrate the jitter contribution in the eye timing margin difference between the SS−40 °C corner (i.e., ~12%) against the fast–fast (FF)−40 °C (i.e., ~5%). Although IO buffer design technology and circuit architecture can be different from the studied case, which may lead to different jitter numbers, considering PGSIJ by the pre-driver is as important as that induced by last-stage P/G rails. It is worth noting that the P/G supply noise of the pre-driver mainly induces jitter distortion, whereas the P/G supply noise of the last-stage inverter introduces both jitter and amplitude distortions, as is illustrated in Figure 6, where both eye plots are compared at the worst-case corner.

### 3.3. Induced Jitter: Shared vs. Decoupled PG Noise

**Figure 7:**
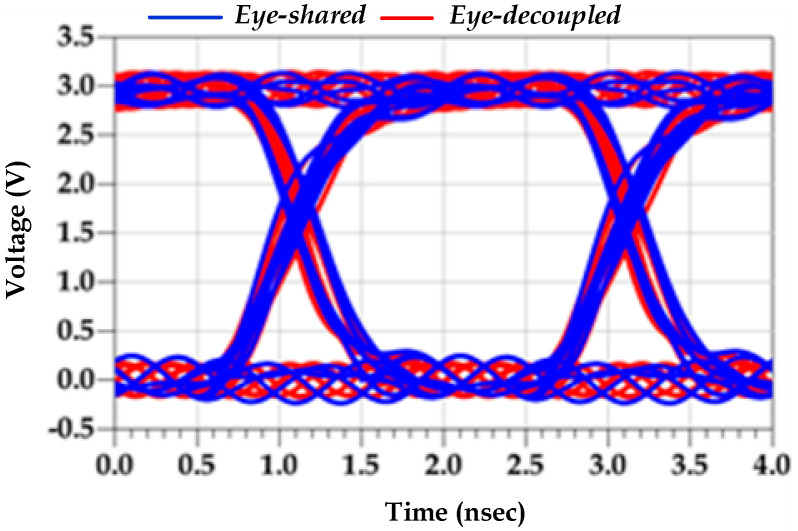
Eye diagram: shared vs. decoupled PGSIJ at SS−40 °C.

The two simulation configurations, which are used to estimate the p2p eye jitter, are shown in Figure 4. The PGSV settings are described in Table 4. The simulation results of both configurations are shown in Table 5. Furthermore, Figure 7 illustrates the eye diagrams of the pre-driver and the last-stage-induced jitter at the SS−40 °C corner.

Table 3 and Table 5 show that the worst-case eye jitter is observed at the SS−40 °C corner for this specific transistor technology and node at which usually the worst jitter performance is observed. Since IO buffers are more sensitive to jitter noise at the SS corner, it is usually recommended to run the high-speed IO link SiPI simulation at the SS corner to determine the timing margins at the receiver’s input. Therefore, the accuracy of the power-aware IO buffer behavioral model should be guaranteed at the SS corner, where it presents the worst-case PGSIJ performance. In addition to that, the p2p jitter for shared and decoupled PG supply noise at the SS corner shows ~53 ps difference, which depends on the PG noise frequency content and amplitude variations.

## 4. PSIJ Sensitivity Study of Two-Stage Driver

### 4.1. Simulation Setup

**Figure 8:**
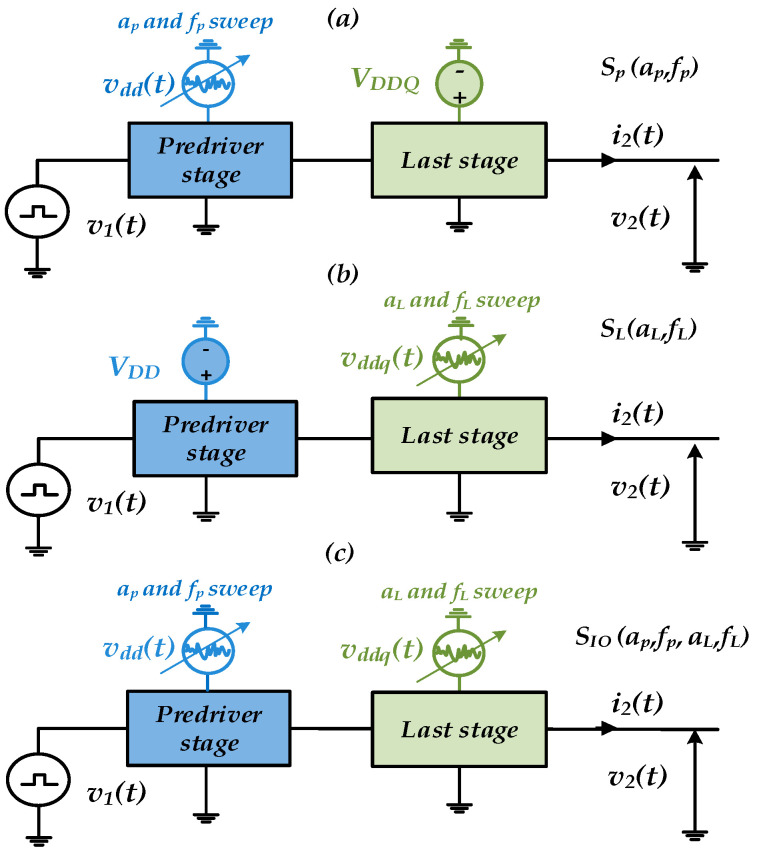
Frequency and amplitude-dependent PSIJ TF characterization (**a**) of pre-driver stage, (**b**) of last stage, (**c**) of driver’s IO stages.

This section aims to explore the study of the sensitivity of the PSIJ transfer function (TF) to the supply voltage amplitude and frequency induced by pre-driver ($V_{DD}$) and last-stage ($V_{DDQ}$) buffer at the SS−40 °C corner [21]. The separate and combined PSIJ contributions of the driver’s IO stages are explored. The PSIJ by the pre-driver $V_{DD}$ supply (i.e., $S_{P}\left(\cdot \right)$) and last-stage supply $V_{DDQ}$, i.e., $S_{L}\left(\cdot \right)$), can separately affect the driver’s total output jitter (i.e., $S_{IO}\left(\cdot \right)$). The PSIJ of the IO device nonlinearly depends on the amplitude (e.g., $a_{k}$) and frequency (e.g., $f_{k}$) of the power supply voltage, as illustrated in Figure 8.

(7) $$PSIJ_{k}\;\left(ps\right)=S_{k}\left(a_{k}\left(V\right),f_{k}\left(Hz\right)\right);\;k=\left\{P,\;L,IO\right\}\;$$

where k indicates the driver (IO) or specific driver stage: pre-driver (P) or last stage (L). The amplitude and frequency of the distinct power supply of the pre-driver and last stage are swept in order to figure out the sensitivity of PSIJ on the frequency and amplitude as shown in Figure 8.

### 4.2. Frequency Sensitivity of PSIJ Transfer Function

**Figure 9:**
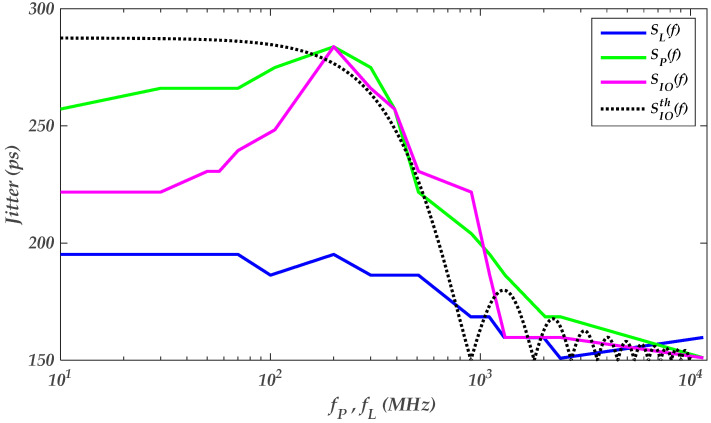
PSIJ experimental and theoretical TF variations due to the separate and the combined impacts of the supply voltage variations of the driver’s IO stages as *a_L_* = *a_P_* = 160 mV.

The last-stage PSIJ is determined by sweeping the frequency of the applied sinusoidal voltage waveform at $V_{DDQ}$, while pre-driver stage supply $V_{DD}$ is kept constant, as shown in Figure 8b. Similarly, the same experiment is performed for the pre-driver stage, as shown in Figure 8a. In the combined PSIJ impact, the distinct supply case of the driver’s IO stages is considered for this experiment, as shown in Figure 8c. Figure 9 shows the PSIJ transfer function of the above three studied cases, as the supply noise frequencies of the separate and combined contributions of the pre-driver and last stage are swept. Table 6 summarizes the supply settings to obtain the PSIJ TF shown in Figure 9.

The comparison, which is shown in Figure 9, between the theoretical jitter TF $S_{IO}^{th}\left(f\right)$ of the IO buffer (e.g., black dashed line curves), which is defined in Equation (5), follows a similar waveform trend as the experimental results of the PSIJ $S_{IO}\left(f\right)$ function. The difference between ($S_{IO}^{th}\left(f\right)$ vs. $S_{IO}\left(f\right)$) at low frequency (e.g., f<100 MHz) can have several explanations. Firstly, the conditions used in the simulation data to derive $S_{IO}^{th}\left(f\right)\;$is a clock signal against a random bit pattern used to derive $S_{IO}\left(f\right)$. Moreover, the deviation between the theoretical and experimental results can be due to the accuracy of the spice model level used in the simulations or the derivation of theoretical function

The PSIJ TF of the pre-driver shows a peak value around 200 MHz. However, the $S_{L}\left(f\right)\;$shows a flat response until reaching 200 MHz. The pre-driver’s jitter is the main contributor to the IO buffer’s total jitter. $S_{p}\left(f\right)$ has a bandwidth of [120 MHz−200 MHz], which defines the $V_{DD}$ signal frequency range that generates the highest PSIJ. As the PSV’s frequency increases and exceeds 200 MHz, the PSIJ number starts to decrease. At higher frequency (f>2 GHz), the PSIJ of both IO driver stages decreases and is dominated by the input data pattern timing distortion.

**Figure 10:**
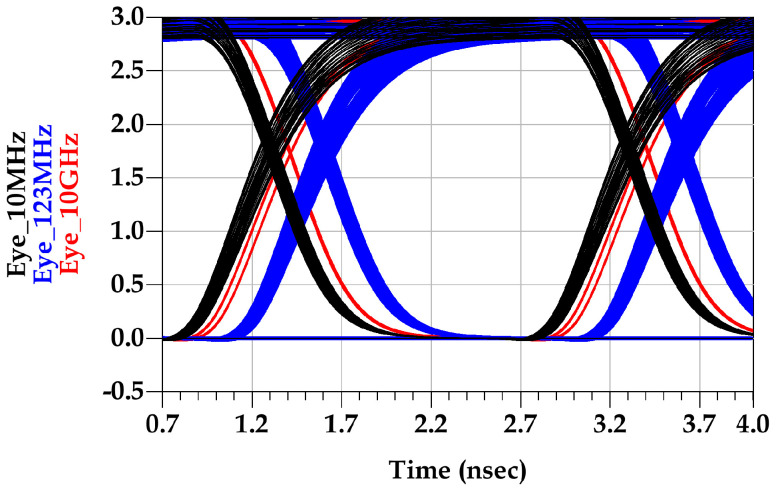
Output eye diagram due to last-stage PGSV variation with $a_{L}=160\;\mathrm{mV}$ at 10 MHz (black), 123 MHz (blue), and 1 GHz (red).

**Figure 11:**
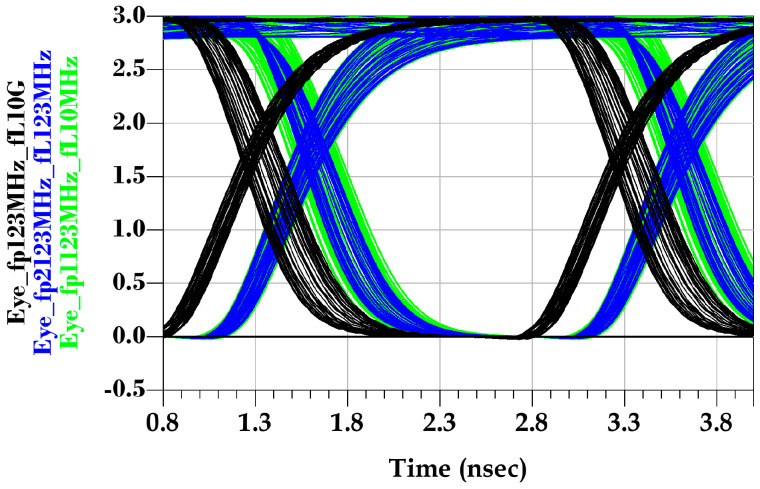
Output eye diagram due to distinct pre-driver and last-stage PSV variations with $a_{L}=a_{p}=160\;\mathrm{mV}$, $f_{P}=123\;\mathrm{MHz}$, while $f_{L}=10\;\mathrm{GHz}$ (black), $f_{L}=$ 123 MHz (blue), and $f_{L}=$ 10 MHz (green).

The eye diagrams shown in Figure 10 demonstrate the PSIJ sensitivity to the frequency of the last-stage supply variations, as the pre-driver-stage supply kept constant. Figure 11 shows the eye diagram sensitivity to the last-stage supply frequency, as the supply voltage variations of the pre-driver is 123 MHz.

It is worth noting that the driver’s total PSIJ jitter $S_{IO}\;\left(f\right)$ depends on the phase difference between the supply voltage variations (e.g., $\varphi_{V_{DDQ}}-\varphi_{V_{DD})}$. This dependency is explored by the second experiment shown in Table 7, where the last-stage frequency of the applied sinusoidal voltage is swept for a different sinusoidal frequency applied at the pre-driver stage 

**Figure 12:**
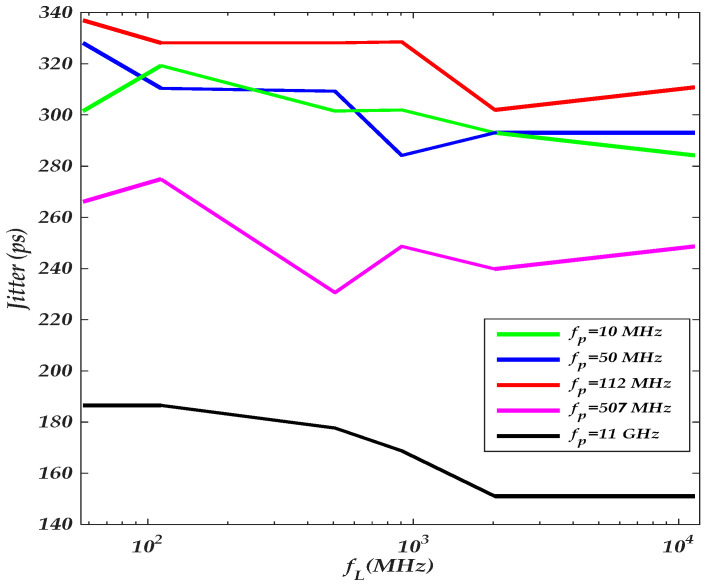
Parametric plot of the driver’s jitter TF as the last-stage frequency is swept for different sinusoidal tone excitation of the pre-driver stage.

As the frequency of the pre-driver supply variations is close the sensitive frequency bandwidth of the $S_{p}\left(f\right)$, the total jitter increases, as shown in Figure 12. Hence, the total jitter at a lower frequency depends on the phase difference between $V_{DD}$ and $V_{DDQ}$ supply voltage waveforms. Moreover, the total jitter is dominated by the pre-driver’s circuit jitter, as the supply voltage frequency of the last stage increases. Therefore, the PSIJ sensitivity can be conceptually assumed to be a superposition of the pre-driver and last-stage PSIJ.

(8) $$S_{IO}\left(f\right)≅S_{P}\left(f_{P}\right)+S_{L}\left(f_{L}\right)-J_{IO}\;$$

where $J_{IO}$ is the jitter induced by the full driver’s input–output timing distortion while the circuit is powered by DC PSV at the SS−40 °C corner.

The superposition of the jitter frequency sensitivity TF can be valid if the supply voltage variation is low enough to approximate the nonlinear I–V and C–V functions of the transistor as linear behavior of the applied input voltage and supply voltage variations. The PSIJ of the pre-driver and last stage can be superposed to lead to the total output jitter if the PGSV (i.e.,$\;V_{DD}$ or $V_{DDQ}$) is composed by two sinusoidal frequency noises under small amplitude variations. The linear superposition of the jitter TF of the pre-driver and last stage can hold under small signal variations. The next section explores how the superposition formula is only valid for small signal analysis.

### 4.3. Amplitude Sensitivity of PSIJ Transfer Function

**Figure 13:**
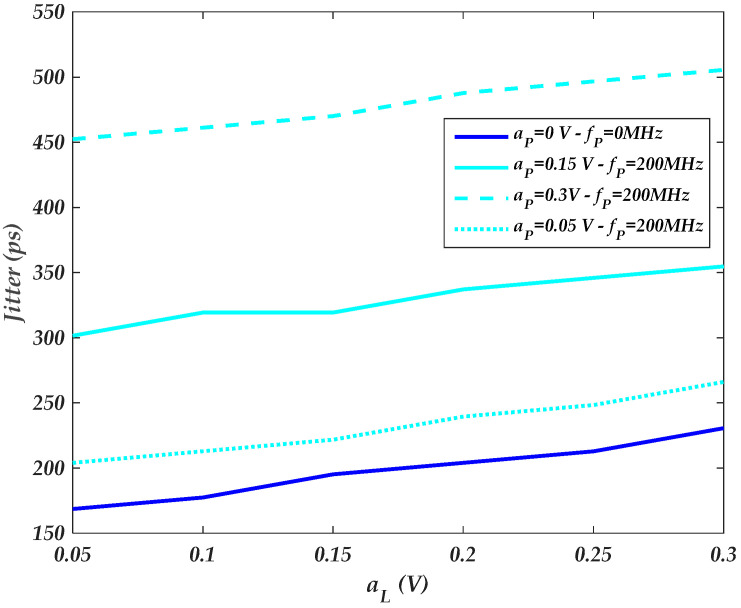
Last-stage parametric plot of the IO buffer amplitude-dependent jitter TF as the last-stage amplitude is swept for different $V_{dd}\left(t\right)\;$ supply noise.

**Figure 14:**
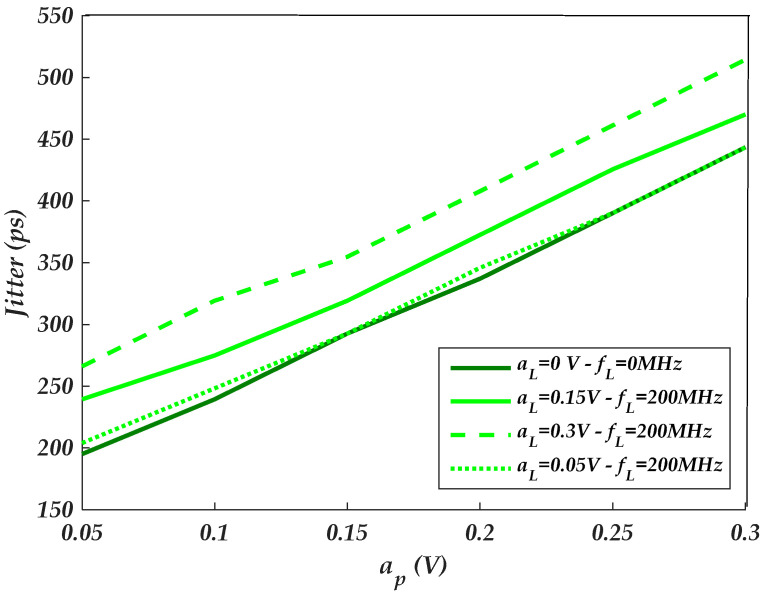
Pre-driver-stage parametric plot of the IO buffer amplitude-dependent jitter TF as the pre-driver stage amplitude is swept for different $V_{ddq}\left(t\right)$ supply noise.

The amplitudes of distinct PSV noises of the driver’s IO-stage circuit are independently swept at f=70 MHz. Figure 13 and Figure 14 show the amplitude-dependent PSIJ of the last stage (i.e., as shown in Figure 8b setup) and the pre-driver stage (i.e., as shown in Figure 8b setup), respectively. Generally, the total IO buffer PSIJ and the separate jitter of the driver’s IO stages increase as the amplitude of the PSV increases.

By comparing the Figure 13 and Figure 14 results, the PSIJ TF has higher sensitivity to the amplitude of the supply voltage noise of the pre-driver than the last stage. This is mainly due the fact that the pre-driver stage is composed of three inverter circuits powered by a $V_{DD}$ supply. For instance, the sensitivity of the pre-driver and last stages are $S_{P}\approx 0.7{\;\mathrm{ps}\;\mathrm{mV}}^{-1}$ (i.e., Figure 14) and $S_{L}\approx 0.12\;\mathrm{ps}{\;\mathrm{mV}}^{-1}$ (i.e., Figure 13).

### 4.4. Two-Tone Jitter Superposition

This investigation aims to validate the jitter superposition condition under small and large-signal distinct PSV variations for both driver’s stages [18,22,23].

#### 4.4.1. Simulation Setting

*Pre-driver case:* Firstly, the pre-driver is simulated for different frequencies with a single tone of amplitude $a_{P}$ of the$\;V_{DD}$ supply voltage, while the last-stage supply is kept constant. Secondly, two tones with different amplitudes (e.g., $a_{P1}$ and $a_{P2}$) are applied at $V_{DD}$, as shown in Figure 8a. The output jitter is measured for both cases and compared against the superposition formula of the output jitter for different amplitude variations.

(9) $$S_{P}\left(f_{P1}+f_{P2}\right)≅S_{P}\left(f_{P1}\right)+S_{P}\left(f_{P2}\right)\;if\;\frac{a_{P1}+a_{P2}}{V_{DD}}\ll 1\;$$

*Last-stage case:* Firstly, the last stage is simulated for different frequencies with a single tone of amplitude $a_{L}$ of the$\;V_{DDQ}$ supply voltage, while the pre-driver supply is kept constant. Secondly, two tones with different amplitudes (e.g., $a_{L1}$ and $a_{L2}$) are applied at $V_{DDQ}$, as shown in Figure 8b. The output jitter is measured for both cases and compared against the superposition formula of the output jitter for different amplitude variations.

(10) $$S_{L}\left(f_{L1}+f_{L2}\right)≅S_{L}\left(f_{L1}\right)+S_{L}\left(f_{L2}\right)\;if\;\frac{a_{L1}+a_{L2}}{V_{DDQ}}\ll 1\;$$

The six cases studied for both IO buffer stages are summarized in Table 8. The frequency of the two-tone supply voltage waveform is selected as a mixture between the low MHz range (e.g., 57 MHz, 123 MHz), where the driver’s IO stage is sensitive to noise, and the higher-frequency range (e.g., 507 MHz, 907 MHz), at which the driver’s IO stage is less sensitive to jitter.

#### 4.4.2. Numerical Results

**Figure 15:**
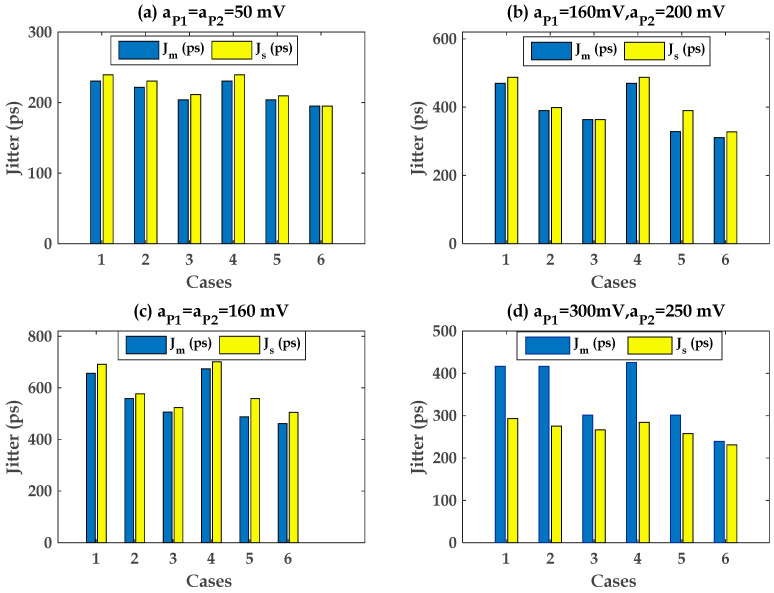
Pre-driver stage case: correlation between $J_{m}$ and $J_{s}$ based on the superposition of (9): (**a**–**c**): small-signal two tones. (**d**): large-signal case.

**Figure 16:**
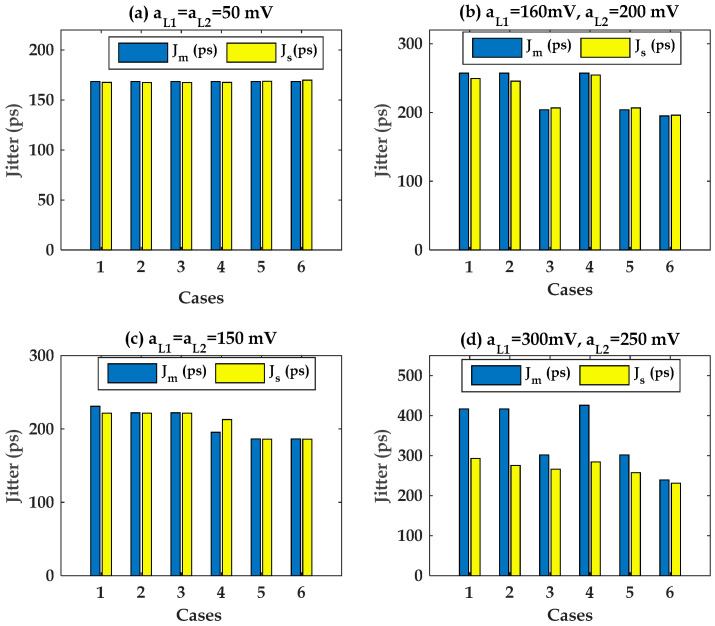
Last-stage case: correlation between $J_{m}$ and $J_{s}\;$ based on the superposition of (10). (**a**–**c**): small-signal two tones. (**d**): large-signal case.

The obtained results of the pre-driver and last stage are illustrated in Figure 15 and Figure 16, respectively. They compare the measured output jitter, $J_{m}$, from transient data against the simulated modelled output jitter, $J_{s}$, of the pre-driver-stage case and last stage using Equations (9) and (10), respectively.

The predicted and measured jitter from simulation data show that a good correlation is observed in Figure 15a–c and Figure 16a–c when the amplitude of the PSV noise is below 25% of the nominal $V_{DD}$ and $V_{DDQ}$ supply voltage. The inaccuracy of the superposition of the PSIJ TF of Equations (9) and (10) is below 4.8% in this case.

As the amplitudes of the PSV variation exceed some specified number, the linear superposition of the applied two-tone voltage contribution of the pre-driver and the last-stage circuit is not valid anymore, as shown in Figure 15d and Figure 16d, respectively. This is mainly due to the fact that the PU and PD transistor current is highly nonlinear, depending on the applied voltage difference between its terminals. The inaccuracy of the superposition of the PSIJ TF of Equations (9) and (10) is below 33.3% in this case.

Cases 1 and 4, where the frequency of the tow tones are a mixture of 75 MHz and 123 MHz, show the highest PSIJ for the studied pre-driver and last-stage cases, as shown in Figure 15 and Figure 16, respectively. This observation confirms the results of the previous analysis which were performed to study the the frequency-dependent jitter sensitivity.

## 5. Conclusions

This paper has studied and analyzed the PGSIJ contribution of the pre-driver and last stage of a cascaded inverter IO buffer at three different PVT corners. The shared and decoupled power and ground supply cases were also investigated and compared.

The contribution of the pre-driver’s PGSIJ can be as important as that of the last stage at the worst corner. Finally, the IO buffers are more sensitive to jitter noise at the SS corner and it is important to develop a specific IBIS-like model to capture the pre-driver’s PGSIJ in order to speed up transient simulation. The impact of the PSIJ TF superposition of a multiple-stage IO buffer powered by distinct power supplies depends on the amplitude of the supply voltage variations. The PSIJ or delay superposition of each stage depends on the amplitude variations where the I–V and C–V functions of the transistor can be linearly approximated.

The findings which are reported by the analyzed and simulated data in this paper urge the improvement of the equivalent circuit IBIS-like and/or parametric curve fitting nonlinear dynamic behavioral modelling methodology in capturing power and signal integrity distortion under separate power and ground voltage variations in order to not only speed up PSIJ characterization, but also to cope with the advance in recent PDN and IO buffer designs. For example, the characterization of voltage–time IBIS data under different PVT corners can be explored to derive an approximation of the PSIJ TF.

## Figures and Tables

**Table 1:** Different PVT corners used to evaluate the PGSIJ via transient simulations of IO buffer circuit configurations.

Corner	$V_{DD}/V_{DDQ}$	T (°C)
SS−40	$-10\%\;V_{DD}{}_{Nom}$	−40
SS125	$-10\%\;V_{DD}{}_{Nom}$	125
SS25	$V_{DD}{}_{Nom}$	25
FF−40	$+10\%\;V_{DD}{}_{Nom}$	125
FF125	$+10\%\;V_{DD}{}_{Nom}$	−40

**Table 2:** PGSV settings of setup Figure 4.

Setup	Setup of Figure 4a,b	
Supply	$v_{dd}\left(t\right)$ $\;\mathrm{or}\;v_{ddq}\left(t\right)$	$v_{ss}\left(t\right)$ $\;\mathrm{or}\;v_{ssq}\left(t\right)$
1st tone	{0.2 V, 800 MHz}	{0.15 V, 1300 MHz}
2nd tone	{0.13 V, 1700 MHz}	{0.18 V, 900 MHz}

**Table 3:** Eye Diagram Metrics: PGSV Noise Sources are Considered at the Pre-driver or Last Stage.

Corner	PG Noise	Jitter p2p (ps)	Jitter/UI (%)	EW (ns)	EH (V)
SS−40 °C	Predriver	239.46	11.97	1.86	2.85
	Last stage	248.34	12.42	1.82	2.51
SS125 °C	Predriver	150.77	7.54	1.89	2.94
	Last stage	133.04	6.65	1.91	2.49
TT25 °C	Predriver	124.20	6.21	1.90	3.25
	Last stage	141.91	7.10	1.90	2.82
FF−40 °C	Predriver	106.43	5.32	1.91	3.61
	Last stage	79.85	3.99	1.95	3.15
FF125 °C	Predriver	133.04	6.65	1.92	3.53
	Last stage	159.65	7.98	1.92	3.16

**Table 4:** PGSV settings of Figure 6 setup.

Setup	Setup of Figure 6		Setup of Figure 6	
Supply	$v_{dd}\left(t\right)$	$v_{ss}\left(t\right)$	$v_{ddq}\left(t\right)$	$v_{ssq}\left(t\right)$
1st tone	{0.2 V, 800 MHz}	{0.15 V, 1300 MHz}	{0.33 V, 1150 MHz}	{0.25 V, 1400 MHz}
2nd tone	{0.13 V, 1700 MHz}	{0.18 V, 900 MHz}	NA	NA

**Table 5:** Eye Diagram Metrics: Shared vs. Decoupled Cases.

Corner	PG Noise	Jitter p2p (ps)	Jitter/UI (%)	EW (ns)	EH (V)
SS−40 °C	Shared	328.50	16.43	1.80	2.50
	Decoupled	275.00	13.75	1.77	2.57
SS 125 °C	Shared	150.78	7.54	1.88	2.51
	Decoupled	195.20	9.76	1.85	2.51
TT 25 °C	Shared	124.17	6.21	1.88	2.83
	Decoupled	186.25	9.31	1.82	2.87
FF−40 °C	Shared	115.30	5.77	1.91	3.17
	Decoupled	133.04	6.65	1.89	3.16
FF 125 °C	Shared	177.60	8.88	1.91	3.15
	Decoupled	150.78	7.54	1.90	3.21

**Table 6:** Experimental settings of the frequency sensitivity of the PSIJ TF of the driver’s IO stages of the results shown in Figure 8.

	Supplies	$V_{DD}$		$V_{DDQ}$	
PSIJ TF		*a_k_* (V)	*f_k_* (MHz)	*a_k_* (V)	*f_k_* (MHz)
$S_{L}\left(\cdot \right)$		0	0	0.16	$\left[10-11\times 10^{3}\right]$
$S_{P}\left(\cdot \right)$		0.16	$\left[10-11\times 10^{3}\right]$	0	0
$S_{IO}\left(\cdot \right)$		0.16	$\left[10-11\times 10^{3}\right]$	0.16	$\left[10-11\times 10^{3}\right]$

**Table 7:** Experimental settings of the frequency sensitivity of the PSIJ transfer function of the driver’s IO stages of results shown in Figure 11.

	Supplies	$V_{DD}$		$V_{DDQ}$	
PSIJ TF		$a_{p}\left(V\right)$	$f_{p}(\mathrm{MHz})$	$a_{L}\left(V\right)$	$f_{L}\left(\mathrm{MHz}\right)$
IO buffer		0.16	$\left[10,\;11\times 10^{3}\right]$	0.16	$\left[57,\;11\times 10^{3}\right]$

**Table 8:** Last stage and pre-driver: frequency settings of Figure 14 and Figure 15.

Cases	$f_{L_{1}},\;f_{P_{1}}\;\left(\mathrm{MHz}\right)$	$f_{L_{2}},\;f_{P_{2}}\;\left(\mathrm{MHz}\right)$
1	57	123
2	57	507
3	57	905
4	123	57
5	123	905
6	123	2033

## Data Availability

Not applicable.

## Abbreviations

**IO**	**Input–Output**
PVT	Process, voltage, and temperature
P/G	Power/ground
PSV	Power supply voltage
PGSV	Power ground supply voltage
PSIJ	Power supply-induced jitter
PGSIJ	Power ground supply-induced jitter
SiPI	Signal and power integrity
PDN	Power delivery network
PCB	Printed circuit board
I–V	Current–voltage
C–V	Capacitance–voltage
IBIS	input–output buffer information specification
TL	Transistor level
SS	Slow–Slow
TT	Typical–Typical
FF	Fast–Fast
TF	Transfer function
p2p	peak-to-peak
EW	Eye width
EH	Eye height
